# Indirect Nutrition and Mobility Risks during Hospitalization: An Architectural Perspective on the nutritionDay Study Findings

**DOI:** 10.3390/nu15061527

**Published:** 2023-03-22

**Authors:** Maja Kevdzija, Alessandro Laviano, Isabella Worf, Christian Schuh, Silvia Tarantino, Michael Hiesmayr

**Affiliations:** 1 TU Wien, Faculty of Architecture and Planning, Institute of Architecture and Design, 1040 Vienna, Austria; 2 Department of Translational and Precision Medicine, University of Rome La Sapienza, 00185 Roma, Italy; 3 Center for Medical Data Science (CeDAS), Medical University of Vienna, 1090 Vienna, Austria; 4 IT-Systems & Communications (ITSC), Medical University of Vienna, 1090 Vienna, Austria

**Keywords:** mobility, nutrition, risk, hospital, hospitalized patients, built environment, hospital ward, architectural design, nutritionDay

## Abstract

Nutrition and mobility risks include complex and interrelated physiological, medical, and social factors. A growing body of evidence demonstrates that the built environment can affect patients’ well-being and recovery. Nevertheless, the relationship between the built environment, nutrition, and mobility in general hospitals is largely unexplored. This study examines the implications of the nutritionDay study’s results for the architectural design of hospital wards and nutrition environments. This one-day annual cross-sectional study uses online questionnaires in 31 different languages to collect ward-specific and patient-specific variables. The main findings relevant to the design of hospital wards were: (1) 61.5% of patients (*n* = 48,700) could walk before hospitalization and (2) this number dropped to 56.8% on nutritionDay (*p* < 0.0001), while the number of bedridden patients increased from 6.5% to 11.5% (*p* < 0.0001), (3) patients who needed more assistance had a much longer mean LOS than mobile patients, (4) mobility was associated with changes in eating, and (5) 72% of units (*n* = 2793) offered additional meals or snacks, but only 30% promoted a positive eating environment. The built environment may indirectly affect hospitalized patients’ mobility, independence, and nutritional intake. Possible future study directions are suggested to further investigate this relationship.

## 1. Introduction

Hospital wards, used for patient recovery, rehabilitation, therapy, and monitoring, are among the hospital’s main functional departments and employ a wide range of personnel [1]. Patients spend the majority of their hospital stay in the wards, and the wards have more floor space than any other hospital department [2]. How a hospital and the individual wards are organized in terms of layout structure, size of spaces, and their connections can greatly affect the behavior and well-being of patients and staff members. Being an inpatient is unpleasant and distressing; patients in a ward environment are likely to be weak, anxious, and have limited control over their environment [3]. Furthermore, malnutrition and decreased mobility are some of the most prevalent issues in hospitalized patients [4], leading to adverse outcomes such as functional decline, a longer hospital stay, the increased necessity for nursing home placement following discharge, and increased mortality risk [5,6,7,8,9]. However, all of the aspects negatively affecting patients’ nutritional intake and mobility during hospitalization are still unclear.

Malnutrition is a common and complex condition that affects many people in an acute hospital setting [10]. To a great extent, malnutrition is caused by either an increase in the number of nutrients the body requires and/or a decrease in food intake due to various factors [11]. Nutrition plays a significant role in every stage of life. Still, it is of utmost significance for older adults, who make up a large portion of hospitalized patients [12] and among whom malnutrition is widespread [13]. The variables contributing to malnutrition are complex and interrelated, including various physiological, medical, and social factors, which should all be considered in treatments to improve nutrition status [14]. The Protected Mealtimes initiative was introduced to improve the nutritional intake in hospitalized patients [15]. Still, very few positive changes have been observed due to its implementation, and the quality of evidence on its effectiveness is low [16,17]. This initiative is often difficult to implement in busy hospital ward environments [18,19], and patients commonly accept frequent mealtime interruptions as an integral part of the hospital environment that cannot be changed [20].

In addition to aging or poor health factors, a decrease in food intake may also be associated with environmental characteristics [21,22,23]. Experience during mealtimes influences the quality of life, independence, feelings of social support, and food intake, especially in older adults [14,24]. Therefore, recognizing that meals provide more than simply nutrients is essential [25]. There are indications that the meal experience can raise patients’ morale, which reduces the daily monotony in a hospital and anchors the day [26]. Additionally, receiving positive encouragement from visitors during mealtimes may increase food consumption [20]. It has also been suggested that eating in a social setting might improve energy intake [27]. Therefore, the environment in which meals are eaten might play a significant role in the malnutrition of hospitalized patients.

During their time in the hospital, patients often struggle not just with malnutrition but also with decreased mobility. Most hospitalized patients rarely leave their beds [28]. Older adults are greatly affected by this, spending over 90% of their hospitalization lying in their beds [28,29,30], which is linked with adverse outcomes [5]. Furthermore, the most typical challenges patients have while transitioning from a healthcare facility to their own homes are those associated with their mobility and the activities of daily living [31]. Therefore, older patients need to exercise more and be mobilized in a hospital to prevent losses in their functional capacity [32]. At the same time, in daily hospital practice, patient mobility frequently fades into the background [32]. Additionally, mobility is commonly restricted in the hospital environment to prevent falls [33].

Barriers to mobility can be complex in a hospital setting. They can include patient symptoms and illness severity, staffing and treatment-related factors, attitudes toward mobility, and environmental factors (such as an inadequate amount of space, equipment, and furniture) [34,35,36]. Safe walking areas, adequate lighting and flooring, communal areas, access to equipment, and functional furniture, were identified by nurses as factors that would facilitate patient mobility [34]. Physicians also highlight that the physical setup of the patient room does not encourage mobility and encourages the patient to stay in bed [35]. Even though the built environment was identified as a mobility barrier, its role in patient mobilization is still greatly understudied in the hospital environment [34]. Rigorous research studies examining the built environment’s influence on patients’ mobility and nutrition are not yet available and are difficult to implement because of the unique character of each facility and the logistical constraints.

This paper examines the architecturally relevant nutritionDay study findings: (1) the structure of the hospital patient population in terms of nutrition and mobility, (2) how their status changed during hospitalization, (3) the relationship between nutrition and mobility, and (4) the availability of an environment that promotes eating. Current research does not consider the role of the architectural ward design in patients’ commonly low nutritional intake and reduced mobility during hospitalization. This research aims to assess the magnitude of the problem and identify the potential of the built environment to support and promote mobility and food intake. Based on the results, we suggest further research directions for investigating the role of the built environment in patients’ decreased food intake and low mobility during hospitalization. 

## 2. Materials and Methods

### 2.1. Research Method

The nutritionDay (www.nutritionday.org, accessed on 15 February 2023) is a global initiative created to increase understanding and awareness of malnutrition in healthcare facilities. It is a one-day annual cross-sectional study repeated yearly since 2006 in hospitals worldwide. Online questionnaires in 31 different languages are used to collect ward-specific and patient-specific variables [37]. Questionnaires are designed to enable the participation of any interested ward; no specialized knowledge or specific laboratory measurements are necessary. Participation after online registration is free of charge. All participating units can download a unit-specific report for auditing purposes compared to units with a similar medical specialty.

The first questionnaire addresses the structure, human resources, and standard nutrition procedures of the ward in which the patient resides and is to be completed with the help of the head nurse or physician. The second questionnaire addresses the caregiver’s view of the patient, including data on the patient’s age, height, weight, medical or surgical condition, comorbidities, and type of nutritional support given. The third questionnaire allows patients to self-report their actual food intake, their self-rated health status, including their mobility level before hospitalization, and their actual mobility status. Patients received help to fill out the questionnaire when needed.

### 2.2. Sample and Data Analysis

Because of the new inclusion of “mobility before admission” in questionnaires, the 2016–2021 nutritionDay database containing data from 49,444 patients was used for analysis. We included all 48,700 patients aged 18 years or above. Unit and patient data are reported as median with interquartile range {IQR} or proportions. Comparison between patient groups with different risk factors was performed with general linear models where the group without risk factors was used as a reference and units were considered as random factors. Association of risk factors with the length of stay before and after nutritionDay was also determined with general linear models with risk factors as categorical variables and units as random factors. All estimates are reported as mean with a 95% confidence interval. All analyses were performed with STATA 15.1.

## 3. Results

### 3.1. Demographics

The analyzed sample includes 48,700 adult hospital patients from 2793 units in 58 countries (Table 1). Twelve countries contributed more than 1000 patients. The regional distribution is as follows: 46% Europe, 4% North America, 26% Latin America, 19% Asia, and 4% Eastern Mediterranean. Sensitivity criteria, which is an 80% outcome recorded and more than six patients included, was fulfilled in 2271/2793 (81%) units. The median unit size was 30 IQR {23–40}, and the actually available beds were 23 IQR {18–31}. Two-thirds of admitted patients were recruited into the nutritionDay study. Seven specialties contributed more than 100 units: general internal medicine 561 (20.1%), general surgery 416 (14.9%), geriatrics 238 (8.5%), oncology 237 (8.5%), gastroenterology 176 (6.3%), orthopedic surgery 118 (4.2%), and cardiology 107 (3.8%).

The median age of the patients was 66 IQR {51–78}, and 49.5% were female. Two-thirds of patients were medical patients and one-third of the total population of surgical patients were either before surgery *n* = 4739 (10%) or after surgery 11,872 (24%) (Table 2). Surgical postoperative patients had been admitted to an intensive care unit before nutritionDay twice as frequently compared with preoperative patients, 2302/11,872 (19%) versus 452/4739 (9.5%) or medical patients 2415/31,189 (7.7%).

The outcome collection 30 days after nutritionDay in the 2271/2793 (81%) units fulfilling the sensitivity criteria of >80% outcome recording and recruitment of more than 6 patients was available in 41,566/41,885 (99.2%) of patients. At outcome collection 30 days after nutritionDay, 30,362/41,885 (72.5%) of patients were discharged home, 4137 (9.9%) were discharged to another care institution, 5071 (11.1%) were still in hospital, and 1342 (3.2%) had died (Table 2).

### 3.2. Mobility, Nutritional Intake, and Well-Being

**Figure 1:**
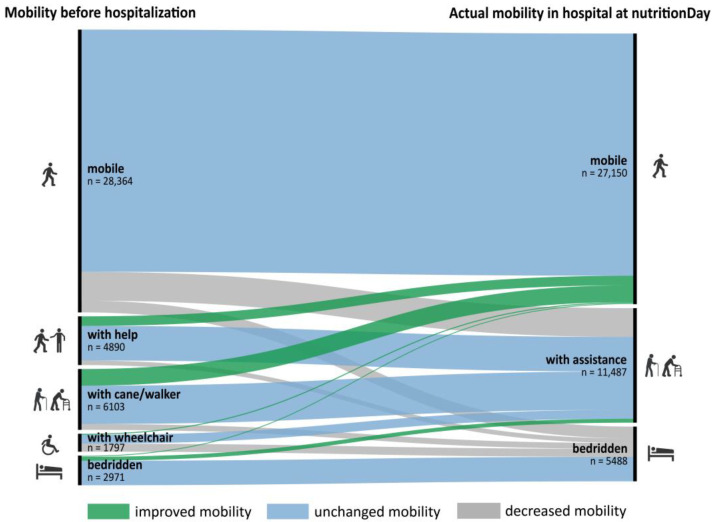
Mobility before hospital admission versus actual mobility at nutritionDay (*n* = 44,125), category “missing” is excluded from the graph.

Mobility was limited for more than a quarter of patients already before hospital admission. When evaluated at nutritionDay, this proportion increased to one-third of patients, with the proportion of patients who were bedridden more than doubling (Table 3). During the period between hospital admission and nutritionDay, a larger proportion of patients indicated an improvement in strength (41.7%) than a decrease in strength (18.3%) (Table 3). Mobility before admission was associated with actual mobility (Figure 1) since about three-fourths of patients who were mobile before admission were also mobile in the hospital and three-fourths of patients who were bedridden remained bedridden. Interestingly, 10% of the previously mobile patients needed help, and 4% were bedridden, whereas 26% of the patients previously needing a cane or help considered themselves mobile within the hospital environment. Between admission and nutritionDay, 12.2% of patients’ mobility worsened (Figure 1).

**Figure 2:**
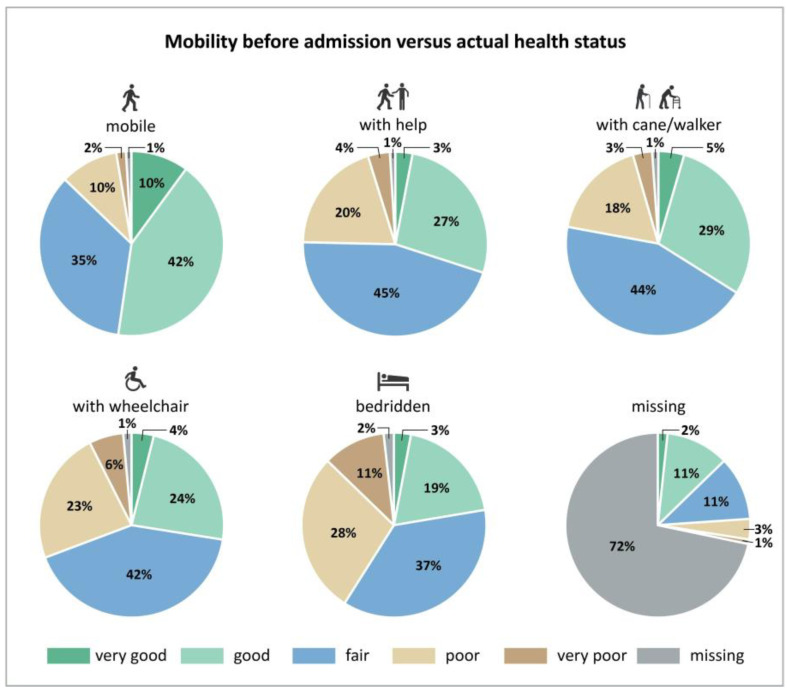
Mobility before hospital admission versus self-reported actual health status at nutritionDay (*n* = 48,700).

**Figure 3:**
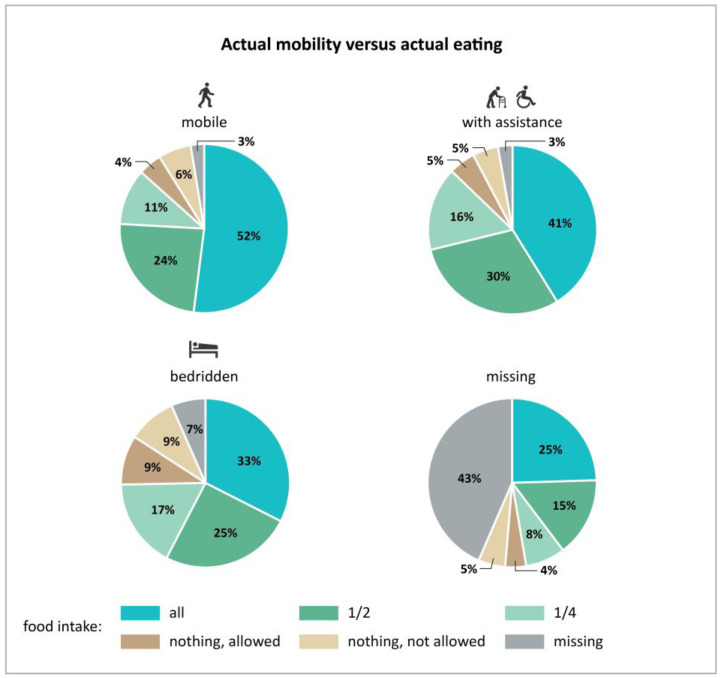
Association between actual mobility and actual eating at a major meal (*n* = 48,700).

**Figure 4:**
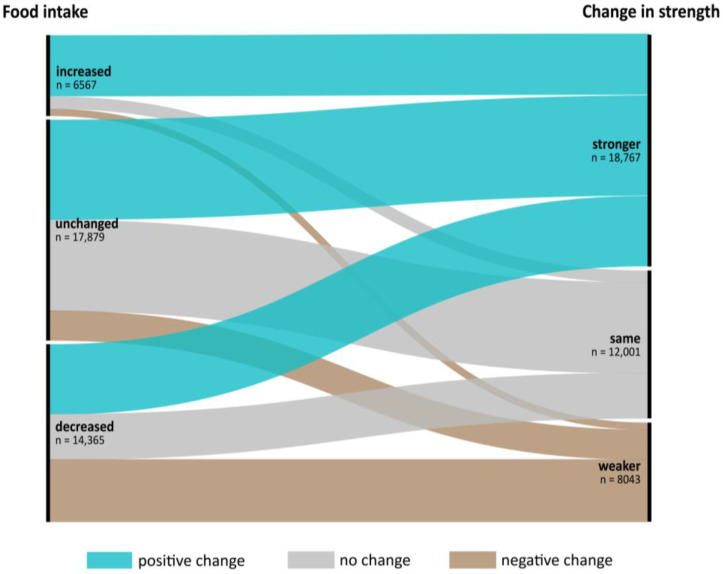
Change of food intake versus change in strength between admission and nutritionDay (*n* = 38,811), categories “I don’t know” and “missing” are excluded from the graph.

Mobility before admission is also associated with self-perceived health. The proportion with “very good” or “good” self-rated health decreased from 52% to 25% with decreasing mobility and “poor” and “very poor” self-rated health increased from 12% to 40% (Figure 2). Eating was not normal before hospitalization in one-third of patients, and not eating their full meal further increased to more than half of the patients. Accordingly, one-third indicated that their eating decreased, while only 14% indicated an increase (Table 4) since hospital admission. Decreased actual mobility was associated with decreased eating (Figure 3) of all the food served. Similarly, patients that became stronger also increased their food intake; it decreased in those feeling weaker compared with patients that did not change their food intake (Figure 4). A large proportion of patients who reported feeling weaker (*n* = 8043) also had a decreased food intake (*n* = 5040) (63%).

Mobility before admission was strongly associated with length of stay between admission and nutritionDay as well as between nutritionDay and discharge (Table 5). Reduced actual mobility and reduced actual eating were both associated with prolonged length of stay after nutritionDay (Table 5). Length of stay after nutritionDay until discharge was more prolonged by impaired mobility by 1.7 (CI95 1.4–1.9) days for those needing help and 2.6 (CI95 2.3–2.9) days for those bedridden, corresponding to an increase of 33% and 50%, respectively. Reduced eating had a smaller effect of 0.35 (CI95 0.16–0.55) days for a half meal eaten and 0.94 (CI95 0.68–1.20) days for a quarter of meal eaten as well as in those eating nothing, 0.93 (CI95 0.55–1.30) days, despite being allowed to eat and was 0.96 (CI95 0.60–1.32) days after adjustment for length of stay before nutritionDay.

**Figure 5:**
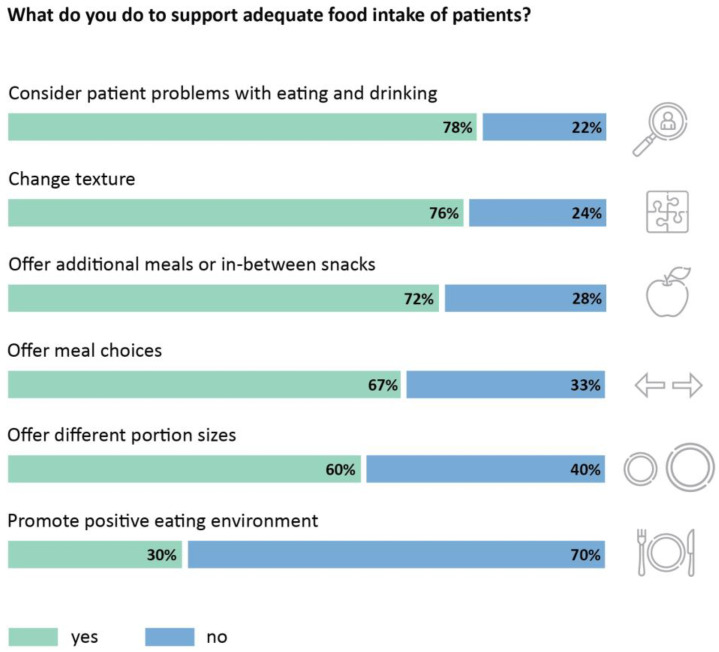
Implementation of strategies that promote a positive eating environment (*n* = 2793 units).

In the unit structure questionnaire, the medical staff was also asked to report on the strategies their unit implemented to support the nutritional intake of patients. The main strategies used in the participating units (*n* = 2793) were offering additional meals or snacks (2012, 72%), offering meal choices (1869, 67%), different portion sizes (1672, 60%), changing texture (2131, 76%), and considering patient problems with eating and drinking (2167, 78%) (Figure 5). Only 828 units (30%) reported promoting a positive eating environment. Furthermore, it was not specified what exactly the term “positive eating environment” entailed.

## 4. Discussion

### 4.1. Main Findings and Their Implications

On nutritionDay, more than half of hospitalized patients reported that they could walk independently, one out of four patients needed assistance for mobility, and 11.5% were bedridden. Even though a large proportion of patients can walk independently, patients are consistently found to spend most of their day lying in bed, with a very low number of covered steps per day [28,29,30]. This creates significant issues, as even short periods of bed rest and low mobility can accelerate muscle loss during hospitalization [5,29,38]. Even the meals are served in bed—the majority of patients have meals at their bedside as the primary location [39,40]. On the other hand, the most successful supportive nutrition interventions in hospital settings, according to systematic reviews, are those that encourage social interaction and communal eating [25,41,42]. Only 45% of patients ate their whole meal on the nutritionDay and around a quarter of patients reported eating apart meals. It is common for family members to bring food in for patients [21]; we also found that 72% of units offered meals/snacks between main meals. These additional meals and snacks are usually eaten at the bedside as well. The patient’s mobility level was also associated with the amount of lunch consumed. Only one-third of bedridden patients ate their complete lunch, and nearly 20% did not eat anything.

In our study, patients unable to walk independently had a much longer mean hospital stay than patients who could walk. Furthermore, 11.1% of patients were still hospitalized 30 days after the nutritionDay. This highlights that patients who need more assistance for mobility, eating, and other ADLs are staying much longer in the hospital, placing a significant burden on the nursing staff. Another important finding is that 18% of patients reported feeling weaker on the nutritionDay compared to admission. Changes in patients’ mobility status during hospitalization are evident: the number of patients who could walk independently dropped from 61.5% before to 56.8% on nutritionDay. At the same time, the proportion of bedridden patients increased from 6.5% to 11.5%. This is not uncommon, as hospital-acquired disability has been observed in other studies [30,43]. Those above the age of 65 are at especially high risk: around a third experience functional deterioration (loss of independence in activities of daily living) after a hospital stay [44]. Older persons hospitalized for non-disabling diseases experience a functional decline both during hospitalization and in the month following discharge due to low mobility and decreased nutritional intake in the hospital [43].

**Figure 6:**
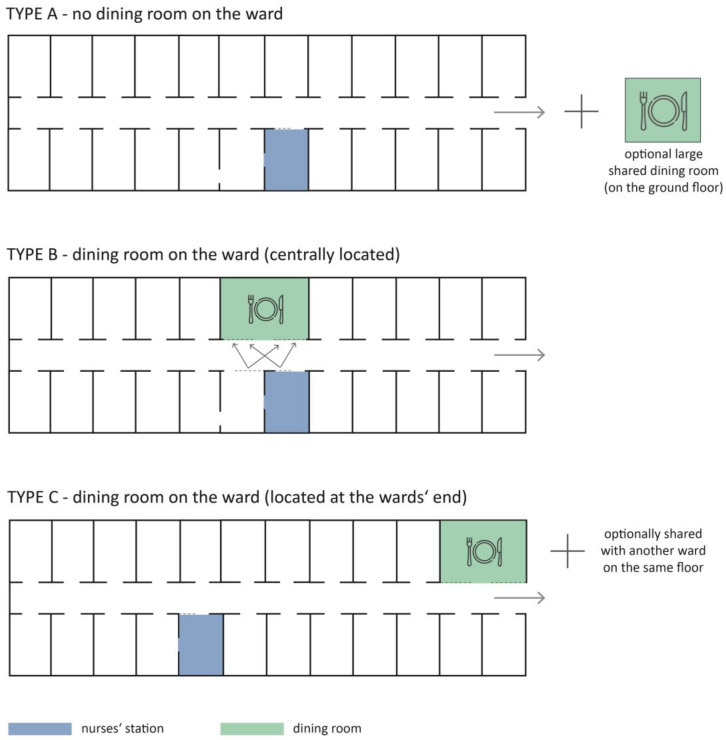
Schematic floorplan representation of the most frequent types of dining rooms in hospitals.

These findings are relevant to the hospital design and, more specifically, ward design. Typical hospital wards often do not have a space where patients can eat outside their rooms, such as a shared dining room, as they are expected to eat at their bedside (Figure 6-Type A). Even when dining rooms exist, they are often underutilized [27,39]. If the room is too far from the patient’s room, it might be difficult for the patient to reach it independently. This creates more workload for the nursing staff to transport patients from their room to the dining room [45]. In a study in the stroke rehabilitation context, the distance was found to play a role in patients’ mobility—patients were only independently visiting spaces close to their rooms [46]. Thus, if communal dining areas for hospitalized patients are implemented, they should be within a distance where most patients can reach them independently. In addition to transporting patients, another challenge of communal eating is keeping careful supervision over all patients during mealtimes, as some patients cannot eat outside their rooms [45]. This could be alleviated by positioning the dining room centrally on the ward and allowing for easy supervision via a large window close to the nurses’ station [45]. This kind of schematic organization is shown in Figure 6 Type B. Another variation in ward layout design has the dining room at the end of the ward, sometimes shared with an adjacent ward (Figure 6-Type C). Other concepts (such as small open seating/dining spaces in the corridors) exist, but they are rarely implemented in hospital design. It is unclear whether any of these options is the best possible solution for dining environments or if a different solution would be more beneficial for the patients.

Because even a limited number of steps taken during hospitalization might mean the difference between functional decline and maintained independence [47], developing mobility- and nutrition-supporting environments may be a joint approach. Mealtimes also show potential to be used as a time to practice skills related to rehabilitation and recovery, such as improving mobility, dexterity, and psychological well-being [27,48]. Where patients eat is important, not only for the main meals of the day but also for snacks and food brought in by family between meals. Patients going independently to eat their meals in the communal dining room could be seen as a mobility exercise, and the dining room could be a place for socialization, which was already shown to improve nutritional intake [25,42]. The location of where main meals are eaten and where snacks are eaten might also not be in the same environment; there could be multiple communal dining options outside of patients’ rooms to allow space to socialize with other patients or to be alone with their visitors. This multiple-space nutritional environment concept needs to be examined from the nurses’ point of view as well, as it would increase the logistical challenges, such as transport and supervision [45].

The role of the built environment in increasing patients’ mobility and activity was already investigated in specific groups, such as stroke patients and patients with dementia. These isolated research studies targeted particular patient groups, and it remains unclear how their findings could be transferred to the general hospital population. Thus, research investigating various environmental interventions to improve general hospital patient mobility is greatly lacking. Some spatial interventions on geriatric hospital units in the form of various environmental interventions [49,50] show the potential in activating patients and improving their mobility levels, but this is still insufficiently researched. Studies investigating the nutrition environment are more numerous but focus on particular elements in the environment, such as the presence of music [51,52,53], the presence of aquariums [54], the use of high-contrast plates and cutlery [55,56], or whether the meals were eaten in the dining room or at the bedside [22,27,57]. Several studies have found that patients/residents who dine in a communal setting benefit from greater nutritional intake, a lower risk of malnutrition, and enhanced socializing [22,58]. Most studies examining nutrition environments were conducted in dementia units in long-term care, not hospitals. Therefore, the hospital nutrition environment design to best support food intake is still an under-researched topic. The presented schematic layout organizations of the wards in Figure 6 are implemented differently in different hospitals around the world and their impact on patients’ food intake and mobility has yet to be evaluated.

When designing hospital wards, our finding that the LOS of patients who are not independently mobile can stretch up to three weeks, on average, needs to be considered. For these patients, the built environment can greatly limit or support their everyday activities in the hospital. At the same time, communal dining can increase the burden on the nurses, who might prefer that all their patients eat in their rooms [45]. Nevertheless, mealtimes are an opportunity for patients to leave their beds and be physically active. If the wards were designed so patients could reach the communal dining environments independently, this would eliminate a significant workload for the nursing staff. Patients using walkers and wheelchairs can still be independently mobile but need specially designed environments that are accessible, easy to navigate, and motivate them to leave their beds. As limited research is available on the topic, it is unclear what threshold distance patients using a wheelchair, cane or walker can cover independently.

### 4.2. Ward and Hospital Design Challenges

Architects are faced with a large variety of rules, guidelines, and criteria when designing hospitals, making the design of hospital wards especially challenging [2]. In addition to the different aspects of patient-centered design that are increasingly being considered, such as privacy, control, and family support, many other factors, including infection control, ventilation, fire safety, patient monitoring, patient transport, staff walking distances, staff communication, and foodservice organization, need to be taken into account when designing ward environments. Furthermore, the development of new technologies (e.g., artificial intelligence and robotics, telemedicine, and health wearables) impacts how the designs of hospitals are developed and future-proofed. Therefore, the design of nutrition environments (e.g., communal dining rooms) or mobility-promoting environments might not be one of the main priorities in hospital ward design.

A survey of 119 architects in the United Kingdom revealed that their main priorities for the ward design were view to the outside, nurses’ observation, access to sanitary facilities, and infection control [2]. In the same survey, social space was ranked 8th in terms of importance among ten design criteria, without specifying what kind of social space it was. Architects without hospital design experience valued social space less [2]. Staff travel distances were ranked 7th, without mentioning patient travel distances or environments promoting patients’ mobility. This study showed that the nutrition and mobility environment design was not mentioned among the top ten priorities for the ward design among the 119 participating architects.

The research on design priorities from other countries is greatly lacking, and healthcare design firms do not readily or openly publish the current design strategies for nutrition and mobility-supporting environments. Nevertheless, architects play a crucial role in creating hospital wards, and their interests can influence and prioritize certain design criteria [2]. At the same time, the care environment, through its architecture and culture, can substantially limit the freedom of both patients and medical personnel to work toward their respective goals [59]. If environments that support the nutritional intake and mobility of patients are not high on the priority list when designing hospitals, this creates an indirect risk for patients, as they will be limited in these aspects during their hospitalization. Therefore, current approaches to designing eating environments in hospitals must be examined in-depth. The same is the case for spatial features supporting the mobility of hospitalized patients.

### 4.3. Future Research Directions

**Figure 7:**
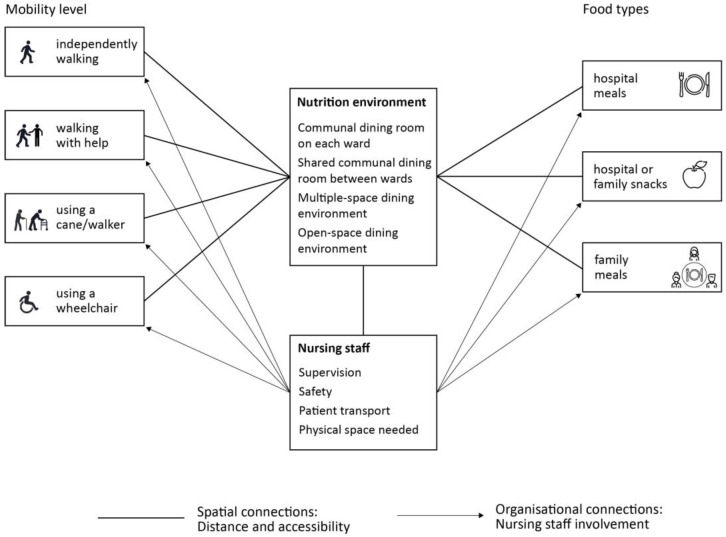
Spatial and organizational connections to be studied in future research.

As the connection between nutrition and mobility in the built environment is still under researched, we suggest potential future research directions based on our study results. Multiple factors interplay in the ward environment (Figure 7); future research could look into the spatial and organizational relationships between the nutrition environment, patients’ mobility levels, food types, and the nursing staff. For example, the distance and accessibility to various types of nutrition environments could be examined to determine what distance and dining room organization would enable most patients to be independently mobile. At the same time, the distance of nursing staff to various nutrition environments needs to be examined to establish the best environment type for nurses’ supervision, safety concerns, patient transport, and the amount of necessary space to provide meals. This also relates to the nursing staff’s involvement in attending meals for patients with different mobility levels. Different food types might require a different type of nutrition environment and a different level of nursing staff involvement.

These proposed connections could be studied both quantitatively and qualitatively. A review of current nutrition environment designs in hospitals worldwide needs to be conducted to establish the existing ward layout organizations. A quantitative research study could compare different layouts with the nutrition environment as a central point, measuring the distance patients need to cover to reach it independently. Barriers to mobility can also be mapped on this path by the systematic observation of patients and nurses. The nutritional intake of patients could be measured for each nutrition environment type and the level of nursing staff involvement. Another potentially promising research direction is the association between the covered distance/daily steps and nutritional intake.

As experimental research introducing real-life new nutrition environment concepts is challenging to implement, VR experiments can be conducted instead to explore the spatial needs of patients and nurses. Another possibility would be to test real-size mock-ups, which would require large empty spaces for setting up. At the same time, more qualitative research is needed to explore the experiences of hospitalized patients, especially patients with low mobility levels with the longest lengths of stay. In addition, Nurses’ experiences and preferences regarding patients’ mobility and independence during mealtimes must be investigated further. We highlight only some options for possible future research into the relationship between the built environment, mobility, and nutrition during hospitalization, as there are many more unexplored topics in this understudied field.

In addition to further examination of how the built environment affects patients’ nutritional intake and mobility during hospitalization, personal and organizational factors need to be considered. Even though low mobility has a major impact and can lead to adverse outcomes, patients’ understanding of the effects of bed rest may be limited to commonly reported symptoms such as pressure ulcers and chest infections [60]. Patients also believe that food is not a priority during hospitalization and that the hospital environment limits their activity [20]. Therefore, when hospitalized, patients expect to lie in bed most of the time and eat less than usual [20]. Educating patients about the importance of mobility and nutrition is crucial for reducing their functional decline during hospitalization. At the same time, the hospital’s organizational culture can greatly influence patients’ activity levels and nutritional intake. If patient mobilization is discouraged on the ward (due to safety or other concerns), this creates another limiting factor that the provision of the mobility-supporting built environment cannot circumvent. Therefore, the nursing staff needs to be educated about supporting the independent mobility of patients during mealtimes, mainly because patient mobilization tends to fade into the background in everyday practice at the hospital [32].

### 4.4. Strengths and Limitations

This study has some potential limitations. Patients with longer lengths of stay in the hospital might be overrepresented due to the cross-sectional research design. The voluntary nature of the survey introduces a selection bias into the study since participating departments are likely to be more interested in nutritional care and adopt more nutritional care interventions. At the level of the patients, a selection based on the severity of the illness cannot be excluded, probably favoring the inclusion of patients in better physical and mental conditions. Another limitation is that food intake measurements were self-reported by patients, which may be less reliable because they are dependent on their individual estimation abilities. Furthermore, the study did not directly examine the impact of various ward design aspects on patients’ mobility and food intake.

The major strengths of this research are the use of the same standardized and simple data collection tool in local languages, the large sample size, the multi-national worldwide participation, and the focus on nutritional care factors.

## 5. Conclusions

Hospitalization often leads to reduced mobility, reduced food intake, and changes in patients’ well-being. These conditions are well known to be associated with poor outcomes. In this study, patients’ mobility conditions worsened compared to before hospitalization. Decreased mobility is associated with reduced food intake and decreased well-being. Patients with more reduced mobility and low food intake also stay in the hospital significantly longer. Of the many strategies adopted by the units to improve patient food intake, promoting a positive eating environment was the least frequently observed. The ward structure, seen from an architectural point of view, might be a key factor influencing patients’ food intake and mobility. Certain ward designs might promote patients’ mobility and nutritional intake, but more studies should be carried out to identify which aspects are more determinant in promoting mobility, food intake, and well-being.

## Figures and Tables

**Table 1:** Unit characteristics (*n* = 2793) from 58 countries.

	Unit	Median	{IQR}
Unit size	beds	30	{23–40}
Actual beds	beds	23	{18–31}
Patients recruited	patients	14	{9–20}
Physicians	number	3	{2–7}
Nurses	number	5	{3–8}
Dieticians	number	1	{0, 1}
Physiotherapists	number	1	{0–3}

**Table 2:** Patient characteristics (*n* = 48,700).

	Unit	*n*	Median {IQR} Percent
Age	year	48,579	66 {51–78}
Gender	% female	24,087/48,700	49.5%
BMI < 18.5	n	3676/46,082	8.0%
BMI > 30	n	8380/46,082	18.2%
Diagnosis (ICD10) +	n	82,816	100%
Digestive		11,757	14.2%
Circulatory		9883	11.9%
Neoplasm		9028	10.9%
Musculoskeletal		7822	9.4%
Respiratory		7369	8.9%
Comorbidities #	n	78,706	100%
Diabetes		10,550	13.4%
Cancer		10,384	13.2%
Cardiac insufficiency		8891	11.3%
Infection		6517	8.2%
Chronic lung disease		5817	7.4%
Duration since admission	days	46,526	7 {3–15}
Duration after nutritionDay &	days	35,326	6 {2–11}
Outcome §	n	41,885	100%
Discharged home		30,362	72.5%
Discharged to another HCF		4137	9.9%
Still in hospital on day 30		5071	11.1%
Death in hospital within 30 days		1342	3.2%

+ 5 most frequent ICD10 diagnostic categories; # 5 most frequent comorbidities; & patients still in hospital at day 30 excluded; and § outcome for units fulfilling sensitivity criteria.

**Table 3:** Mobility before admission and at nutritionDay as well as the self-reported health status and evolution during hospitalization (*n* = 48,700).

	*n*	Percent
Mobility before hospitalization		
Mobile	29,939	61.5
With someone‘s help	5168	10.6
With a cane/walker	6463	13.3
With a wheelchair	1930	4.0
Bedridden	3168	6.5
Missing	2032	4.2
Mobility at nutritionDay		
Mobile	27,683	56.8
With assistance	11,713	24.1
Bedridden	5623	11.5
Missing	3681	7.6
In general, how would you say your health is?		
Very good	3652	7.5
Good	17,199	35.3
Fair	17,783	36.5
Poor	6608	13.6
Very poor	1400	2.9
Missing	2058	4.2
Feeling today compared with admission		
Stronger	20,238	41.6
Same	13,337	27.4
Weaker	8913	18.3
Admitted today	1223	2.5
I do not know	2228	4.6
Missing	2761	5.7

**Table 4:** Eating before hospital admission, at nutritionDay, and self-reported change in nutrient intake (*n* = 48,700).

	*n*	Percent
How did you eat the week before hospital admission		
More than normal	2221	4.6
Normal	28,642	58.8
¾	4991	10.2
½	5974	12.3
¼ or less	4383	9.0
I do not know	388	0.8
Missing	2101	4.3
Eating on nutritionDay		
All	21,911	45.0
Half	12,077	24.8
Quarter	6082	12.5
Nothing (allowed to eat)	2517	5.2
Nothing (not allowed to eat)	3024	6.2
Missing	3089	6.3
Change in food intake since admission		
Increased	6856	14.1
Unchanged	19,381	39.8
Decreased	15,260	31.3
I do not know	3076	6.3
Missing	4127	8.5
Eating apart meals		
Yes	12,391	25.4
No	30,354	62.3
I do not know	504	1.0
Missing	5451	11.2

**Table 5:** Eating on nutritionDay and length of stay after nutritionDay.

	*n*	Median (days)	IQR (days)
Mobility before hospitalization and duration since admission			
Mobile (ref)	28,738	5	{3–12}
Mobile with help	4899	9 *	{4–18}
Mobile with cane/walker	6238	9 *	{4–18}
Use wheelchair	1800	11 *	{5–24}
I am bedridden	2987	11 *	{5–24}
Missing	1496	8	{3–17}
Mobility before hospitalization and length of stay after nutritionDay			
Mobile (ref)	22,347	5	{2–9}
Mobile with help	3619	7 ***	{3–13}
Mobile with cane/walker	4802	7 ***	{3–13}
Use wheelchair	1239	8 ***	{3–16}
I am bedridden	2047	8 ***	{4–15}
Missing	1064	6 ***	{2–13}
Actual mobility at nutritionDay and length of stay after nutritionDay			
Mobile (ref)	20,789	5	{2–9}
Mobile with assistance	8401	7 ***	{3–13}
I stay in bed	3772	8 ***	{4–15}
Missing	2276	6 ***	{2–13}
Eating on nutritionDay			
All (ref)	16,270	5	{2–10}
Half	8638	6 ***	{2–12}
Quarter	4332	6 ***	{2–13}
Nothing (allowed to eat)	1817	6 ***	{3–12}
Nothing (not allowed to eat)	2223	6 ***	{3–11}
Missing	1958	6 ***	{2–13}

* significant difference in length of stay before or after nutritionDay between reference and individual risk factor categories (*p* < 0.01), *** (*p* < 0.0001) in GLM estimation.

## Data Availability

The nutritionDay project provides original data to researchers submitting a protocol that needs to receive approval from the scientific committee and acceptance of the data sharing agreement. All information is available at www.nutritionday.org (accessed on 15 February 2023).
